# Experimental Study on Monitoring Damage Progression of Basalt-FRP Reinforced Concrete Slabs Using Acoustic Emission and Machine Learning

**DOI:** 10.3390/s23208356

**Published:** 2023-10-10

**Authors:** Tonghao Zhang, Mohammad Mahdi, Mohsen Issa, Chenxi Xu, Didem Ozevin

**Affiliations:** Department of Civil, Materials, and Environmental Engineering, University of Illinois Chicago, 929 West Taylor Street, Chicago, IL 60607, USA; tzhang91@uic.edu (T.Z.); missa@uic.edu (M.I.); cxu43@uic.edu (C.X.)

**Keywords:** basalt-FRP, acoustic emission, bridge deck, SHAP analysis, unsupervised machine learning, supervised machine learning

## Abstract

Basalt fiber-reinforced polymer (BFRP) reinforced concrete is a new alternative to conventional steel-reinforced concrete due to its high tensile strength and corrosion resistance characteristics. However, as BFRP is a brittle material, unexpected failure of concrete structures reinforced with BFRP may occur. In this study, the damage initiation and progression of BFRP-reinforced concrete slabs were monitored using the acoustic emission (AE) method as a structural health monitoring (SHM) solution. Two simply supported slabs were instrumented with an array of AE sensors in addition to a high-resolution camera, strain, and displacement sensors and then loaded until failure. The dominant damage mechanism was concrete cracking due to the over-reinforced design and adequate BFRP bar-concrete bonding. The AE method was evaluated in terms of identifying the damage initiation, progression from tensile to shear cracks, and the evolution of crack width. Unsupervised machine learning was applied to the AE data obtained from the first slab testing to develop the clusters of the damage mechanisms. The cluster results were validated using the k-means supervised learning model applied to the data obtained from the second slab. The accuracy of the K-NN model trained on the first slab was 99.2% in predicting three clusters (tensile crack, shear crack, and noise). Due to the limitation of a single indicator to characterize complex damage properties, a Statistical SHapley Additive exPlanation (SHAP) analysis was conducted to quantify the contribution of each AE feature to crack width. Based on the SHAP analysis, the AE duration had the highest correlation with the crack width. The cumulative duration of the AE sensor near the crack had close to 100% accuracy to track the crack width. It was concluded that the AE sensors positioned at the mid-span of slabs can be used as an effective SHM solution to monitor the initiation of tensile cracks, sudden changes in structural response due to major damage, damage evolution from tensile to shear cracks, and the progression of crack width.

## 1. Introduction

Reinforced concrete is one of the most common composite structural systems for constructing buildings, bridge girders, bridge decks, and dams. While concrete has exceptional durability compared to other materials, minor cracks may cause the penetration of water and the corrosion of conventional steel reinforcing bars. Fiber-reinforced polymer (FRP) bars are alternatives to steel reinforcing bars as they are corrosion resistant and lightweight. The alternative polymer materials are basalt, glass, carbon, and aramid. Basalt fibers are made from igneous volcanic rocks melted at 1400 °C using a technology similar to that used to produce glass and carbon fibers. The basalt fiber reinforced polymer (BFRP) bars have high tensile strength which is comparable to glass fiber-reinforced polymer (GFRP), while often being more cost-efficient than the carbon fiber-reinforced polymer (CFRP) [1]. GFRP, while affordable, can degrade over time due to the alkaline environment in concrete [1,2,3]. BFRP balances good tensile strength with commendable corrosion resistance, making it a compelling choice to replace conventional steel reinforcement. Due to their high tensile strength, good chemical resistance, and environmental friendliness, BFRP bars have great potential to solve the corrosion problem, especially for bridge decks where deflection is not an issue. However, they are brittle materials with a linear stress–strain behavior up to failure. Nanni [4] and Bank [5] illustrated that the failure of BFRP bars is far more dangerous than concrete crushing. Understanding the mechanisms of concrete failure and detecting them at their earliest stage can prevent unexpected failures, such as detecting and repairing micro-cracks before turning them into macro-cracks. Structural health monitoring (SHM) of BFRP reinforced concrete decks can detect the initiation of microcracks in concrete before any failure occurs at BFRP bars and provides additional reliability in their structural performance.

SHM methods can be adapted to structures for identifying, locating, and sizing defects. Typical SHM methods applied to concrete structures are vibration [6,7,8], strain monitoring [9,10,11,12,13], and the acoustic emission (AE) method [14,15,16,17,18,19]. The AE method is a passive nondestructive evaluation method that detects elastic waves released from active defects. The method can be applied short term for monitoring critical locations or long term for monitoring global behavior; in certain applications, the AE sensors are continuously maintained over extended periods of time, typically 24/7, spanning several years. This approach classifies the method as SHM. Understanding the characteristics of the AE signal during the failure is essential to identify the concrete integrity. AE features, such as hits, cumulative energy, and rise time, are used to determine crack initiation and progression [20,21,22,23]. The crack modes, such as tensile and shear cracks, are classified using rise time (RT)—average frequency (AF) analysis [15,18,24,25]. The statistics-based b-value analysis has also been proven to have a reliable conclusion on the damaged condition of the concrete material [14,15,18,26]. The b-value in the time frame can reflect the concrete responses under the damage. 

Due to the inherent heterogeneous characteristics of concrete, it is difficult to use a single AE feature to evaluate and predict concrete damage evolution. It is necessary to consider multiple AE features, although the relationship between AE features and damage is very complex. SHapley Additive exPlanations (SHAP) is an integrated framework for interpreting model prediction, expressing the importance of each feature for a specific prediction. Lee et al. [27] conducted SHAP analysis to determine the contribution of each AE feature to the rock damage severity. The results indicated that the AE cumulative absolute energy and AE initiation frequency are the most contributing factors significantly impacting the rock damage. 

While the AE method has been applied to both reinforced concrete [23,28,29,30] and fiber-reinforced polymer-strengthened concrete [31,32,33], there have been limited studies on applying the AE method to BFRP reinforced concrete. Chen and Chen [34] studied AE characteristics corresponding to the different stages of the BFRP concrete bond-slip process using small coupons. Most studies reported in the literature were conducted on small-scale reinforced concrete beams. To the best knowledge of the authors, this study is the first time that AE monitoring as an SHM solution has been implemented in BFRP reinforced bridge decks.

In this paper, the AE data collected from two large-scale concrete slabs with different BFRP reinforcing bar sizes were trained to detect damage evolution and characterization. The slab size was comparable to short-span bridge decks with dimensions of 3.05 m in length, 1.22 m in width, and 0.2 m in depth. Typical AE features adapted to reinforced concrete structures, including AE hit rate and amplitude, were evaluated. The damage initiation can easily be determined using the cumulative AE hits in the laboratory environment as the load gradually increases. In an actual application, the slab is under varying loading. A feature-based unsupervised machine learning algorithm was trained to classify each AE hit as representing friction emissions (i.e., background noise, loading, etc.) and tensile/shear cracks using the data obtained from the first slab. The repeatability of the machine learning algorithm was tested using the data obtained from the second slab. SHAP analysis was conducted to investigate the sensitivity of each AE feature to the crack width as an effective AE feature differentiation tool compared to conventional graphical correlation plots. It was shown that the AE sensors positioned at the mid-span of slabs can be used as an effective SHM solution to monitor (i) the initiation of tensile cracks using the machine learning developed in this study, (ii) sudden changes in structural response due to major damage, shifting of clusters from tensile crack to shear crack, and (iii) the progression of crack width by tracking cumulative AE duration using the sensor at the vicinity of the crack. To the best knowledge of the authors, this is the first study in the literature to implement AE in two BFRP reinforced slabs in large scale and apply SHAP analysis to determine the most correlated AE feature with a physical quality (i.e., crack width).

## 2. AE Features for Damage Evolution and Characterization in Concrete

**Figure 1:**
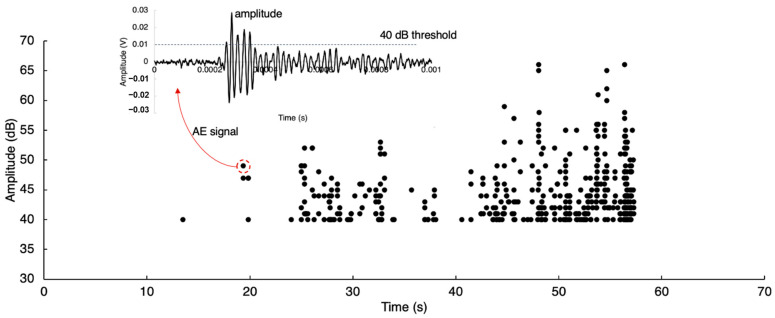
Example of AE amplitude extracted from an AE signal. The red arrow shows the time history signal of the AE hit with an amplitude near 50 dB.

AE features are extracted from time history AE signals to monitor the damage initiation and correlate them with the AE sources. Figure 1 shows AE amplitudes recorded from the first 60 s of the slab experiment using a 40 dB threshold, highlighted at 0.01 V level. The other time domain features extracted from AE signals include rise time, decay time, energy, count, average frequency, and duration [35]. The time history signal is converted into the frequency domain to extract frequency-dependent features, namely peak frequency, and frequency centroid. The AE features are used for identifying the damage initiation and modes. The cumulative energy and count are typically selected to determine the initiation time of damage, and they have been successfully demonstrated in the laboratory conditions where the loading condition is controlled. The cumulative AE features over time may not be valid in a field application due to the variation in loading. The discrete derivative of cumulative energy is also applied to understand AE energy due to the damage evolution [36]. 

The damage modes in concrete are identified using cluster analyses of two or more AE features in the multivariant domain. Shear and tensile cracks are separated using RA (depending on the rise time and amplitude), and AF (average frequency) scatter plots. RA is calculated by the ratio of rise time and amplitude. AF is defined as the ratio of AE counts and duration. The tensile crack appears with a shorter rise time and a larger amplitude during the fracture process, such that a lower RA value is obtained. Shear crack has a longer rise time and lower amplitude. The data points above the pre-defined diagonal line indicate the tensile crack, whereas the part below represents the shear crack. RA-AF analysis is adaptable to small-scale experiments [37]. However, the results from large scale concrete samples (over 0.5 m) show that they are unreliable due to the influence of wave attenuation. The wave attenuation leads to a larger RA value and smaller AF value, which misleads the classification results [38,39]. Unsupervised cluster analysis is a data-driven method that categorizes data points into clusters by grouping data points that possess similar characteristics or features. The AE signals are categorized by the similarity in their characteristics [39,40,41]. This study investigated the feature-based—counts, energy, absolute energy, rise time, initiation frequency, peak frequency, frequency centroid, amplitude, and duration selected to represent each AE signal—damage detection and classification methods using machine learning methods for monitoring BFRP-reinforced slabs with a reduced number of AE sensors to minimize the SHM cost. The results show the importance of structural design and behavior in interpreting the AE results. 

## 3. Experimental Design

### 3.1. BFRP Concrete Slabs

Two slabs with different BFRP bar sizes summarized in Table 1, were constructed and tested monotonically until failure. The slab thickness of 0.2 m was kept constant. The slabs were designed and loaded for wheel point loading. The concrete was made of a typical concrete mix design for bridge deck slabs in Illinois, air-entrained with 6% and a slump of 15.24 cm (6 inches) with a water–cement ratio of 0.445. To prevent the damage caused by movement and experiment setting up, the concrete slabs were cast-in-place [42]. The average concrete compressive strength was measured using 15 cm × 30 cm (6 in. × 12 in.) cylinders at about 44.8 MPa (6500 psi).

Sand-coated BFRP bars, composed of 75% fiber content set in vinyl ester resin, were utilized as primary reinforcement [42]. The tensile properties of #5 and #6 BFRP bars are presented in Table 2. BFRP bars exhibit a linear stress–strain tensile behavior until failure with no yielding point. In general, in concrete design with steel reinforcement, the reinforcement ratio needs to be less than the balanced condition to improve the ductility of the structural element. However, in FRP material, the material design requires a higher reinforcement ratio than balanced conditions to prevent the failure of any FRP material. As the slabs were designed as an over-reinforced section, primary sources of the AE signals were expected due to concrete cracking. 

### 3.2. Instrumentation

**Figure 2:**
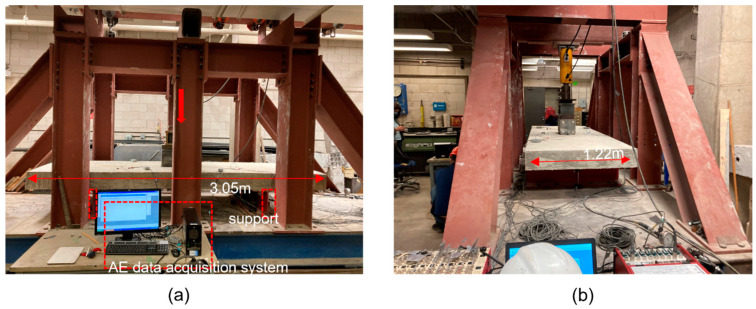
Photos of experimental setup: (**a**) slab testing from front orientation, (**b**) slab testing from a side orientation. The red arrow indicates the position of load cell that can be seen in the photo of side orientation.

**Figure 3:**
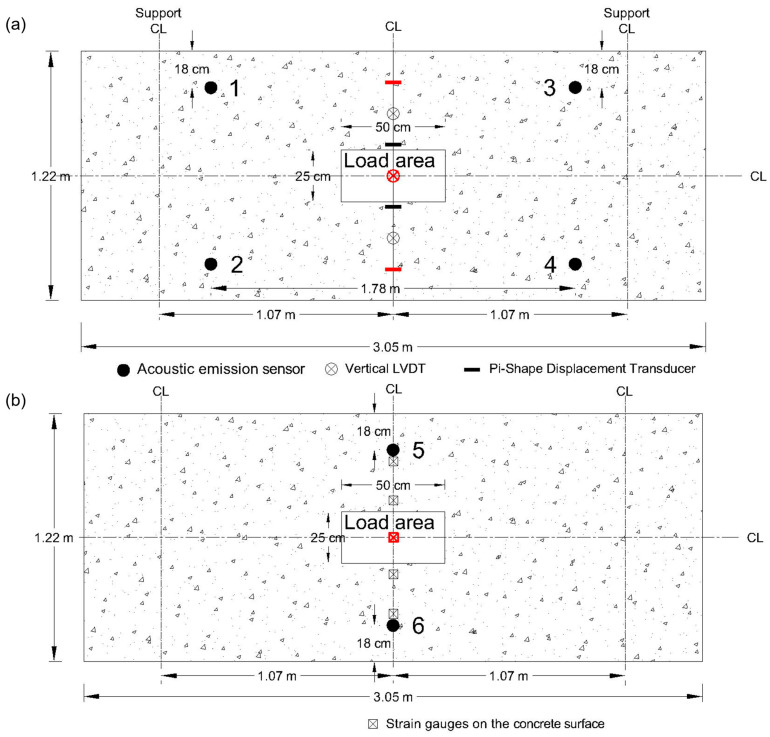
Sensor positions: (**a**) bottom surface, (**b**) top surface. LVDT and strain sensors used to analyze AE data are highlighted in red.

The slabs were loaded from one point using hydraulic actuators as shown in Figure 2. The slabs were instrumented with AE sensors, LVDT (linear variable differential transformer), crack meter, strain gauges, and video camera. The AE sensors, LVDT, crack meters, and strain gauges were attached to the concrete surface. Six AE sensors were distributed on each slab, and their positions are shown in Figure 3. Four sensors were positioned close to supports, and two sensors were placed at the mid-span. To ensure that the selected sensor placement can cover the concrete slab, the pencil lead break testing was carried out prior to the testing at a distance away from the sensor, with an approximate 10 cm increase in the distance. The signal amplitude reaches 40 dB, typically the threshold used in AE testing, about 120 cm away from the sensor. Therefore, six sensors sufficed to cover the slab without being excessive. The sensors were coupled to the concrete surface using hot glue. R6 sensors with an operating frequency range of 35–100 kHz were selected. As concrete is a heterogeneous and attenuative material, this is a typical frequency range used in large scale concrete structures to increase the sensor coverage area. Each sensor was connected to an external preamplifier with a 40 dB gain, and the AE data were collected with a PCI-8 data acquisition system. Sensors, preamplifiers, and AE data acquisition systems were manufactured by MISTRAS Group Inc. The data acquisition variables were 40 dB threshold, 1 MHz sampling rate, and 20 kHz to 400 kHz analog filter. The sensor coupling was checked using the pencil lead break testing about 10 cm away from each sensor. The average and standard deviation values of AE amplitudes for slab 1 were 69.7 dB and 1.7 dB, respectively. The average and standard deviation values of AE amplitudes for slab 2 were 70.9 dB and 2.1 dB, respectively. 

The parameters for hit definition time, hit lockout time, and peak definition time were fixed at their default values, which are 1000 μs, 1000 μs, and 200 μs respectively. Hit definition time determines the end of the AE signal (no more threshold crossing within the window of the hit definition time). Hit lockout time is employed to restrict the system from recording new AE signals, thereby reducing the chance of classifying reflected AE signals as new hits. Peak definition time is utilized to identify the maximum amplitude of an AE signal. While the AE sensors were positioned for developing two-dimensional source localization, the heterogeneity of concrete and the formation of multiple simultaneous cracks caused a complex data set for applying the conventional location algorithms [43,44]. Therefore, this study focused on individual sensor-based analysis to determine the damage initiation and progression. 

The slabs were instrumented with three vertical LVDT sensors positioned in the mid-span to measure the deflection of the slab. The pi-shaped displacement transducers were placed to monitor the crack-width growth at the bottom surface of the concrete, illustrated as crack meters in Figure 3. The strain gauges and LVDT sensors in the loading area, highlighted in red in the figure, were chosen because they provide a more accurate and representative reading than the others. The outputs of LVDT sensors, crack meters, and strain gauges were used to validate the AE results for the damage initiation and modes. 

## 4. Experimental Results

The AE data were evaluated to understand the earliest stage of crack initiation, the identification of damage modes using machine learning methods, and the AE features representing the crack width using SHAP analysis. While these values (i.e., crack initiation, damage mode, and crack width) can be measured in the laboratory environment through strain and displacement sensors at discrete points providing local information, the AE method provides an alternative as a non-intrusive solution for field deployment as a global monitoring approach. 

### 4.1. The Identification of Damage Progression Using Global Analysis

**Figure 4:**
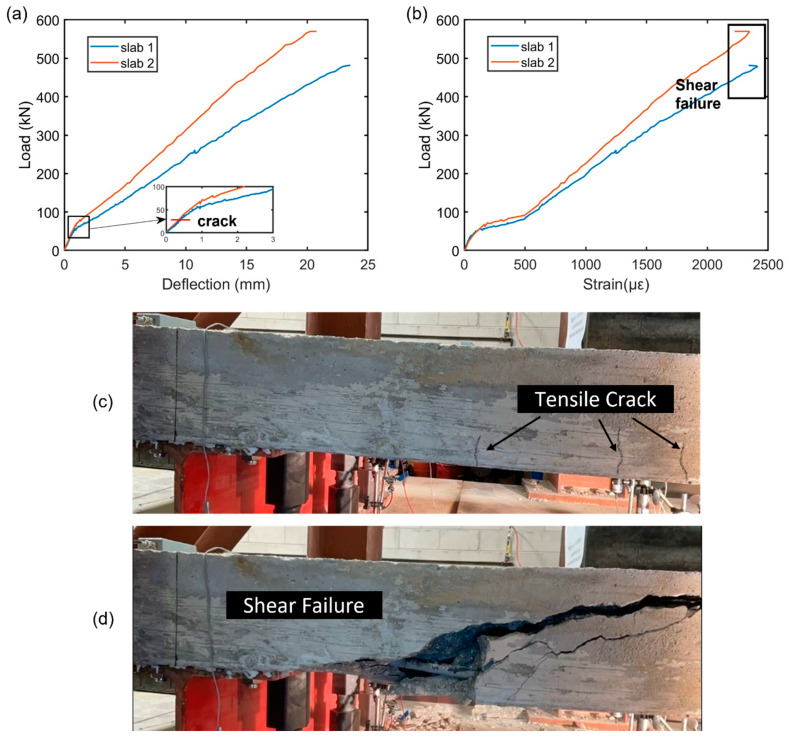
(**a**) The evolution of load as a function of deflection, (**b**) the evolution of the load as a function of strain, (**c**) the captured image when tensile cracks initiated, (**d**) shear crack formed before the failure.

During the loading process, each slab underwent damage. After the tensile stress exceeded the modulus of rupture of concrete, the tensile crack began to form at the positive moment region as shown in Figure 4c.

The time of the first crack development in the positive moment region was evaluated by the load value corresponding to the bending initiation in the load–deflection curve obtained by LVDT as shown in Figure 4a. The major shear rupture was determined by observing the shear crack formation near the support before shear failure as shown in Figure 4d. This can be verified by observing the sudden drop of the compression strain by the end of the load–strain curve as depicted in Figure 4b. The load–deflection curves of slab 1 and 2 reveal similar patterns in the zone before the crack starts, indicating that the bar size does not affect the tensile crack formation. The proportionality of the load–deflection curve becomes steeper in slab 2 due to the increased reinforcement ratio, resulting in a larger load required to produce the same amount of deformation. Similarly, the load–strain curve varies significantly with the reinforcement ratio, exhibiting a stiffer curve as the reinforcement ratio increases. 

### 4.2. The Identification of Damage Progression Using AE Feature Analysis

**Figure 5:**
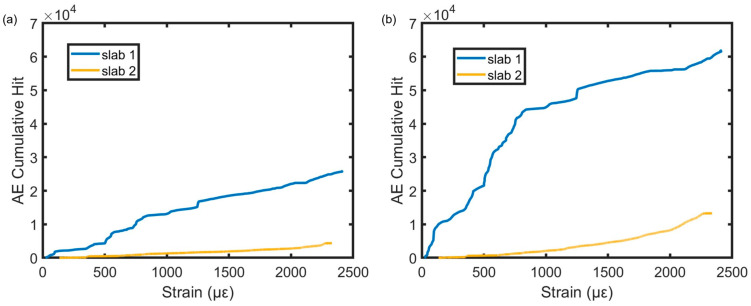
AE cumulative hit versus strain until failure for the two slabs at (**a**) the support collected from sensor 1 and (**b**) mid-span collected from sensor 5.

The correlation between AE features and local strain response offers an alternative means of monitoring local damage when direct strain data are not available. In practical scenarios where strain sensing is absent, identifying the damage status using AE becomes valuable. By investigating the correlation between AE features and strain, the relationship between AE signals and the underlying damage in concrete can be leveraged. Using the data recorded by one sensor placed near the support and at the mid-span of both slabs with varying reinforcement ratios, a comparative study was carried out on the variance of AE hit with respect to strain level throughout the loading testing until concrete failed, as shown in Figure 5. The first slab with the lower reinforcement ratio had higher AE cumulative hit than the second slab in both the support and mid-span regions. Increasing the reinforcement ratio reduced the AE hits due to the higher stiffness in the structure after the first tensile cracking stage. Figure 5 reveals that the sensor at the mid-span detected more AE activities due to their proximity to the mid-span region with the highest deflection. 

**Figure 6:**
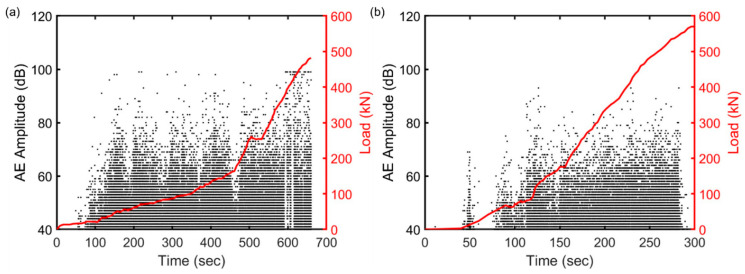
The AE amplitude distribution of sensor 5 (representing the mid-span region) with the increasing load (**a**) slab 1 and (**b**) slab 2. The red line shows the applied load, and each black dot shows the amplitude of AE hit.

As shown in Figure 6, the observed behavior of AE activity changed in response to the applied load, leading to a rise in the amplitude of AE hits associated with the initiation of the crack until reaching its peak when the major crack started to develop. It can be seen that the time interval depicted for slab 1 is considerably longer compared to slab 2. This discrepancy arises from the fact that both concrete slabs are over reinforced, and increasing the reinforcement ratio can adversely affect the ductility, resulting in a shorter duration time for slab 2. It is important to acknowledge that sensor 5 (located near the loading area) and sensor 1 (situated close to the support) are likely to capture certain noise signals resulting from activities in the loading area and support movement. Subsequently, in the upcoming section, a classification method based on machine learning was employed to effectively differentiate between the noise signals and the actual crack signals.

### 4.3. The Identification of Damage Modes Using Machine Learning

**Figure 7:**
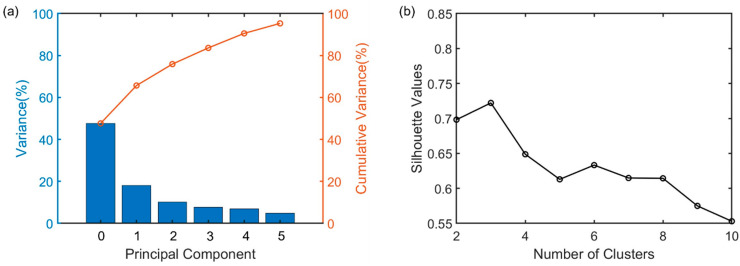
The selection of the number of principal component axes and clusters within the AE data set. (**a**) The variance considered by a varying number of principal components and (**b**) determining the optimal cluster number of the data set using the Silhouette coefficient based on the first 5 selected principal components.

Differentiating AE sources with distinct characteristics, such as rise time, frequency centroid, and peak frequency is achieved by the clustering method, which is based on separating the AE sources by grouping the AE signals with similar characteristics. The most common clustering method is k-mean cluster analysis, that divides a dataset into k distinct, non-overlapping subsets. It iteratively reassigns the data points to the nearest centroid and recalculates the centroid, ensuring the convergence to a solution. The k-mean method is particularly suitable for large datasets, taking advantage of its computation efficiency and simplicity. Herein, k-mean cluster analysis was implemented to fingerprint the AE clusters (e.g., noises/friction, tensile crack, and shear crack) following the training and the testing of data collected from slab 1 and slab 2, respectively. As the AE features depend on the source–sensor distance, the algorithm was designed for one sensor (sensor 5) as the training set. The dataset was selectively chosen in order to minimize the impact of a single cluster dominating the majority of the signals. The first 180 s of data (noted as 20 s after the tensile crack appears observed in the load–deflection curve) and the last 20 s of data (mainly the shear crack) were selected to apply unsupervised machine learning. The algorithm was further validated by utilizing the sensor 6 data, which was also placed close to mid-span. Before conducting the cluster analysis, three preprocessing steps were applied. First, nine AE features, namely, counts, energy, absolute energy, rise time, initiation frequency, peak frequency, frequency centroid, amplitude, and duration, were selected to represent each AE signal. Then, the AE features were normalized with unit variance to reduce the influence of feature units in the cluster analysis. The unit variance normalization addresses the heterogeneities of units in the data and aligns with best practices for handling diverse units in the principal component analysis. The final preprocessing step, as depicted in Figure 7a, involved selecting the appropriate number of principal component axes (PCA) that effectively captured the majority of the variance in the dataset while preventing overfitting. Five principal components carrying more than 90%  variance were considered in the study. 

The last step before cluster analysis was to determine the ideal number of clusters within the data set by the silhouette value, which is employed to evaluate the agreement of an object with the other elements in its own cluster compared to those in other clusters [45,46,47]. The silhouette value for each datapoint is calculated as (b-a)/max(a,b), where ‘a’ represents the average distance to other points in the same cluster, and ‘b’ represents the average distance to points in the nearest neighboring cluster. The value ranges from −1 to 1, the higher the value is, the better the model fits into the corresponding cluster value. As indicated in Figure 7b, the model exhibits the maximum performance when three clusters are considered in the dataset. 

**Figure 8:**
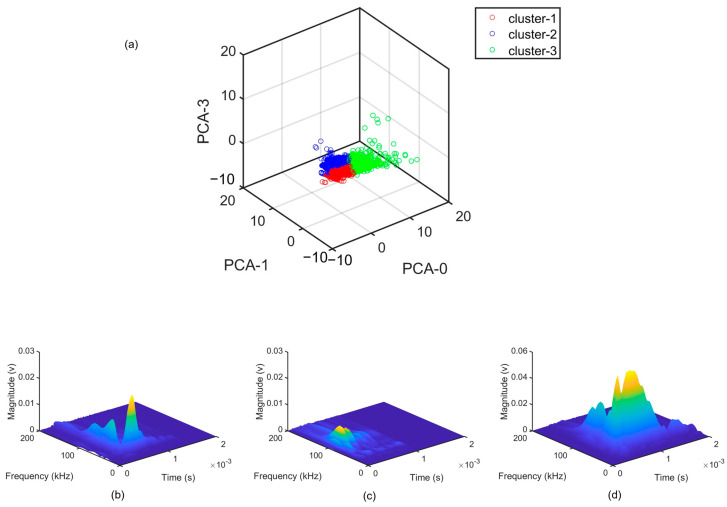
Cluster results: (**a**) Distribution of three clusters in PCA axes and examples of spectrogram of waveforms within each cluster; (**b**) cluster-1, (**c**) cluster-2, (**d**) cluster-3.

Figure 8 shows three distinct clusters representing the AE sources within the data set. PCA-0 is selected as the *x*-axis, and PCA-1 and PCA-3 are selected as the other axes to achieve the best separation and optimal visualization. The clusters possess varying characteristics regarding both time and frequency features, as indicated in Figure 8b–d. 

**Figure 9:**
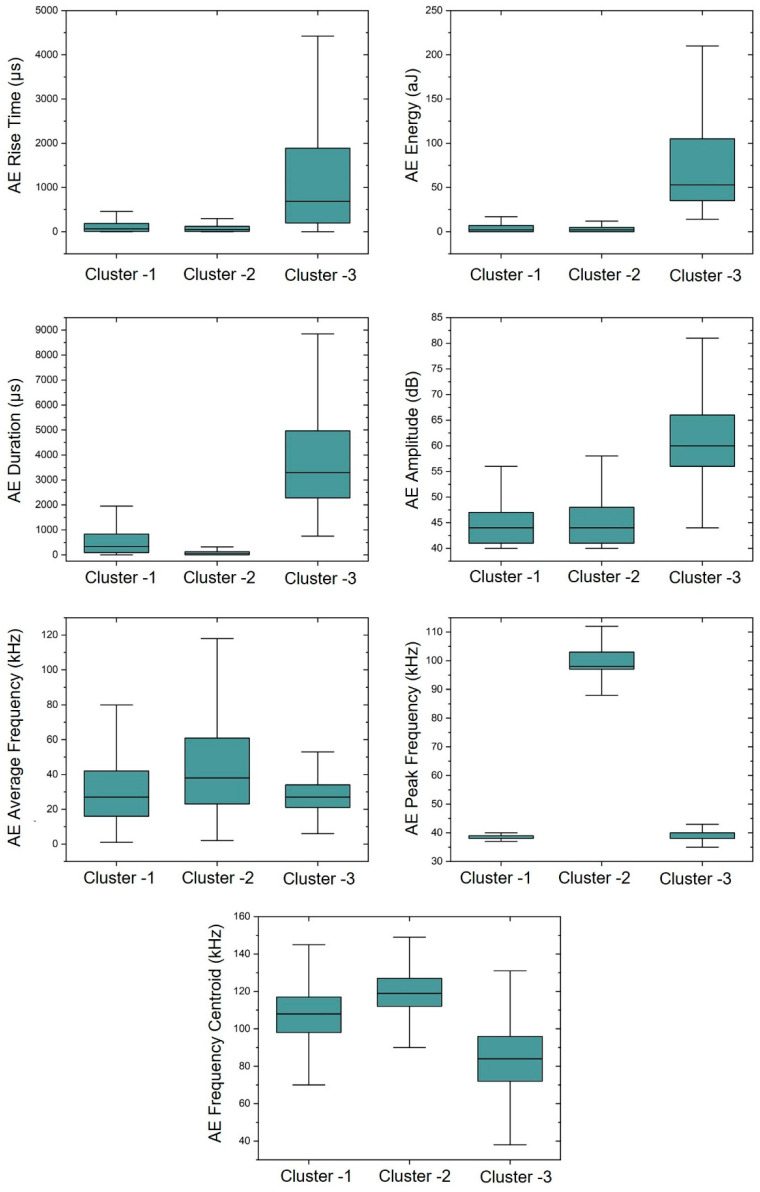
The mean and standard deviation of AE features within each cluster using the sensor 5 data of slab 1.

Table 3 and Figure 9 summarize the mean values of the AE features and distribution of data within each cluster. In Figure 9, the boundaries in the box plot represent the 25th percentile and the 75th percentile, indicating the range within which the majority of data points lie. The center line within the box represents the median value, which is the mid-point of the data distribution. The lines extending from the top and bottom of the box are the maximum and minimum value within each cluster. Cluster-1 is the most active data, representing 66% of total AE hits. Cluster-2 has the smallest AE energy and duration while exhibiting the highest AE peak frequency and frequency centroid among the three clusters. Cluster-3 is the least active source, representing 7.5% of total AE hits. Cluster-3 has the highest AE amplitude, energy, duration and rise time, as well as the lowest average frequency and frequency centroid, which aligns with the characteristics of shear crack in the literature [48,49,50]. The AE feature comparison presented in Figure 9 demonstrates the effectiveness of utilizing the AE features to differentiate the AE clusters. For example, AE rise time, energy, and duration can serve as effective indicators for identifying the cluster-3 while the AE peak frequency can be utilized to differentiate the cluster-2 signal in the AE analysis. It is important to note that the absolute AE features shown in Table 3 are controlled by the AE sensor, threshold, and sensor–source distance. However, the relative behaviors of clusters are expected to be similar for different configurations. 

**Figure 10:**
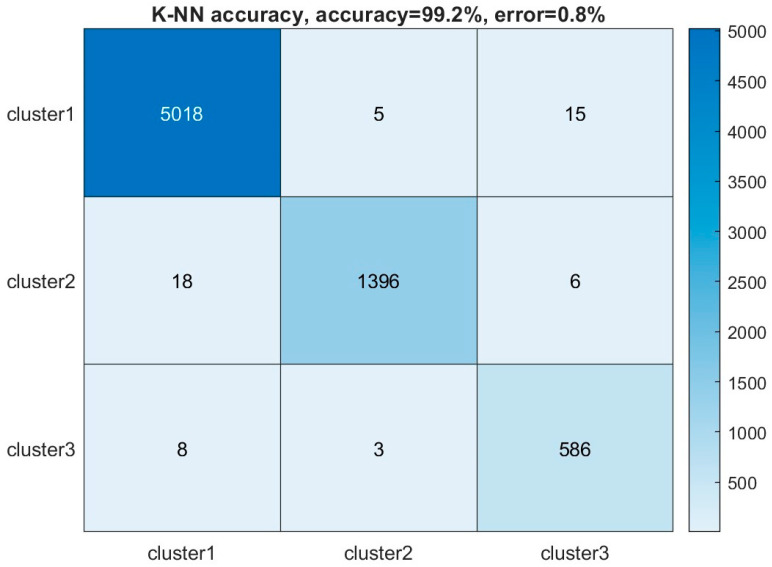
K-NN accuracy of predicting the labels of different clusters obtained from the sensor 5 data of slab 1.

To better understand the relationship between each cluster and its corresponding AE source and evaluate the robustness of this clustering model, a supervised machine learning method using the k-nearest neighbor (K-NN) method [51] was applied to anticipate the cluster label of each AE hit. The data set was divided into half for training and testing subsets. The dissimilarity between each data point was calculated using the Euclidean distance metric because it requires less computational cost, especially in the real time monitoring that collects an extensive dataset. The unit variance scaling and PCA dimensionality reduction in the preprocessing steps would also enhance the performance of the K-NN model when using the Euclidean distance. In addition, to determine the number of neighbors to be selected in the K-NN model, we evaluated different quantities of neighbors. Selecting three neighbors provides the highest accuracy of 99.2%. In contrast, when five neighbors are selected, the prediction error increases to 21.9%. The decision based on one nearest neighbor might not be representative of the overall data distribution, making it susceptible to outliers. Therefore, the number of neighbors was selected as three in the study. The K-NN model’s accuracy is demonstrated by comparing the predicted labels to the actual labels in Figure 10. The values in the non-diagonal positions represent the confusion matrix. The majority of AE signals are classified accurately in their assigned clusters. 

**Figure 11:**
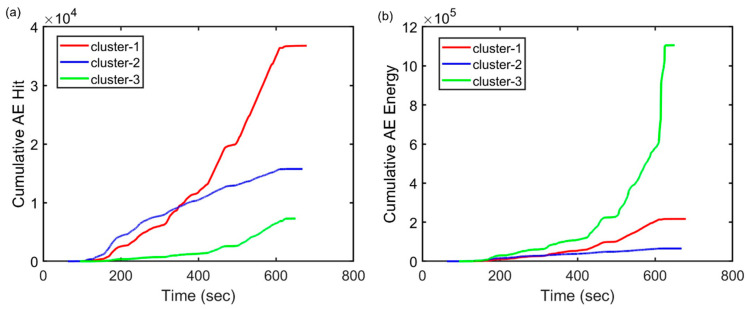
Variation in cumulative AE features of the clusters obtained from the sensor 6 data of slab 1. (**a**) AE cumulative hit and (**b**) AE cumulative energy.

The machine learning model was validated using sensor 6 data, positioned at the first slab’s mid-span. Figure 11a,b demonstrates the cumulative AE hits and the cumulative AE energy of three clusters, respectively. Cluster-2 appears throughout the testing, displaying the lowest AE energy and shortest duration, consistent with the characteristics of artificial noise sources such as friction and loading noise. The cumulative hit of cluster-1 follows cluster-2 with a moderate release of AE energy, a relatively high frequency, and a longer duration than cluster-2. This cluster is considered to represent the tensile crack. Cluster-3 appears later in the loading stage and has significantly high AE energy, which is considered to represent the shear crack.

**Figure 12:**
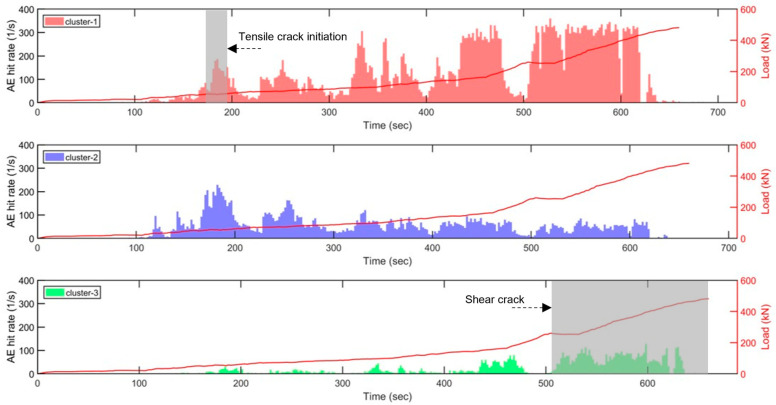
AE hit rate and load versus time of each cluster obtained from the sensor 6 data of slab 1. Grey boxes in clusters 1 and 3 indicate high volume of AE hits detected, which corresponds to the times that the initiations of tensile and shear cracks are visually detected (highlighted with an arrow). The solid red line shows the applied load.

Figure 12 depicts the variation in the AE hit rates of three clusters of sensor 6 with respect to load over time for slab 1. The time step of the AE hit rate histogram is selected as 1 s. Cluster-1 exhibits a minor variation in the first 160 s, followed by a sudden increase. The substantial increase in AE coincides with the initiation of tensile cracks in concrete, as observed in the load–deflection curve, thereby indicating that cluster-1 is related to tensile cracking as noted above. A similar AE hit rate variation pattern is evident in cluster-2, with earlier AE activity occurring at approximately 100 s and continuing throughout the loading. Major AE activity in cluster-3 occurs much later in the loading process, prior to the major fracture event, representing the shear crack. 

**Figure 13:**
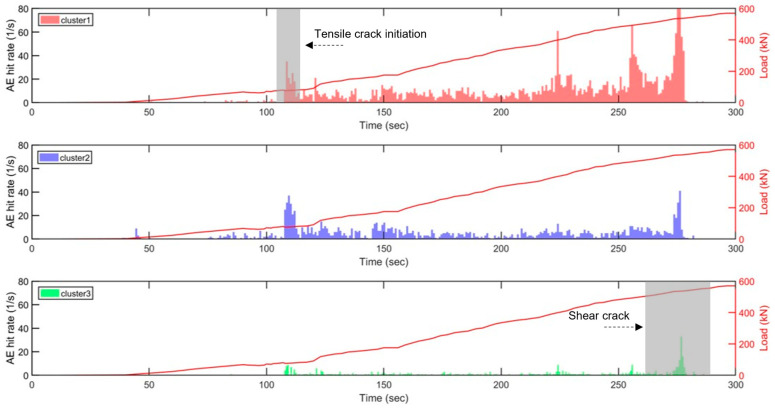
AE hit rate and load versus time of each cluster obtained from the sensor 5 data of slab 2. Similar to the slab 1 result, grey boxes in clusters 1 and 3 indicate high volume of AE hits detected, which corresponds to the times that the initiations of tensile and shear cracks are visually detected (highlighted with an arrow). The solid red line shows the applied load.

The machine learning model was further applied to the slab 2 data recorded by sensor 5. As shown in Figure 13, the number of AE events classified as cluster-1 (tensile crack) begins near the 110 s mark, consistent with the initiation of the tensile crack. Cluster-2 (noise) is observed with a similar hit rate throughout the testing. Cluster-3 (shear crack) occurs close to the end of testing as expected. As both concrete slabs are over reinforced, a higher reinforcement ratio negatively impacts the concrete’s ductility, leading to a shorter duration between the start of shear cracking and structural failure. As a result, the findings from cluster 3 indicate a shorter window of time for taking precautionary actions before structural failure. 

As a summary, Table 4 presents the classification result of the proposed machine learning model. The majority of the dataset is occupied by cluster-1, representing tensile cracks, which consistently appear in both of the two testing sets. In contrast, cluster-3, the shear cracks, occur exclusively before failure and demonstrate consistent presence in both of the testing sets.

### 4.4. SHAP Analysis for the Correlation of AE Feature and Crack Width

When the crack width reaches 0.3 mm, it is classified as a structural crack that leads to a decrease in the structural capacity [52]. In this study, the crack width was directly measured by a crack meter. The aim was to determine the most sensitive AE feature regarding the crack width such that the AE method can be used to predict the crack width if the crack is located in similar proximity to the AE sensor. As shown previously in Figure 3, AE sensors 5 and 6 were placed in close proximity to the crack meters. The spacing between the two types of sensors was the thickness of the concrete (0.2 m).

SHAP analysis was implemented to quantify the contributions of the selected AE features to the crack width. The SHAP value is based on the game’s theoretically optimal Shapley values. The SHAP method explains the prediction of an instance by computing each feature to the prediction. To quantify the contribution of individual features, the SHAP value is calculated by first examining all possible combinations of features. It then measures how much the prediction changes when the feature of interest is included versus when it is excluded. The model ensures the feature contributions are fairly allocated, adding up to the total change in prediction. This helps to understand the distinct role each feature plays in the prediction. A detailed conceptual explanation of SHAP can be found in [53]. Herein, eight primary AE features (counts, absolute energy, rise time, initiation frequency, peak frequency, frequency centroid, amplitude, and duration) were selected as variances. The model input was the discrete variation of each AE feature with time, and the crack width was the model output. 

**Figure 14:**
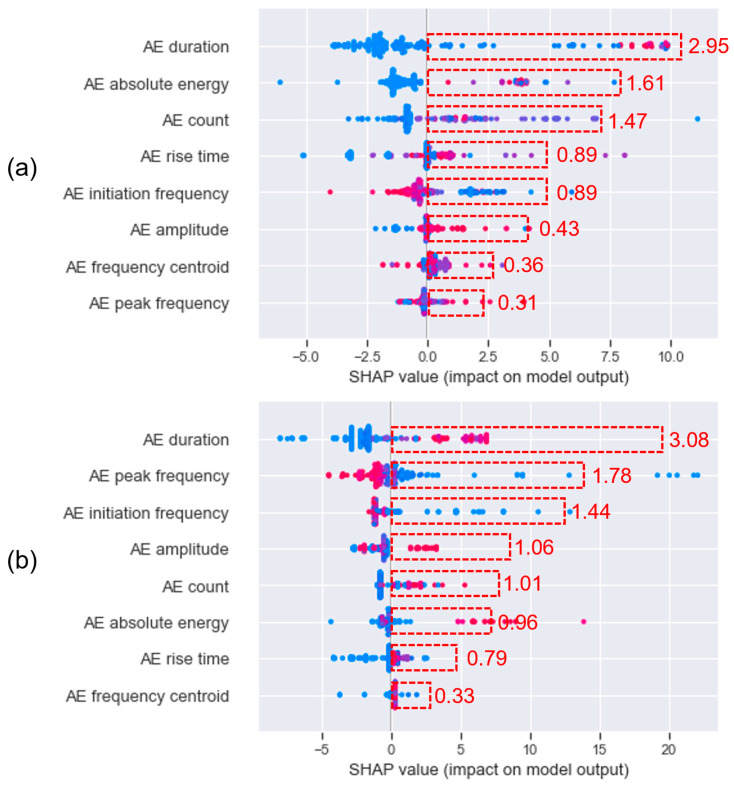
Distribution of SHAP values for AE features. The higher the SHAP value, the higher is the correlation of the AE feature to the crack width growth. The color scale in the SHAP plot signifies feature value magnitude—red for high, blue for low. The red dashed box indicates the mean values of the absolute SHAP values of AE features. (**a**) Sensor 5 and (**b**) sensor 6.

Figure 14 shows the SHAP values of the selected AE features. A higher SHAP value indicates a higher positive contribution to the output (i.e., crack width), whereas a lower SHAP value represents a higher negative contribution to the output. Each point in the SHAP plot represents a single instance in the dataset, and the color represents the value of the specific feature for that instance relative to their range—red for high and blue for low. The point with a positive SHAP value indicates that the value of that feature for this instance has a positive effect on the prediction and vice versa. The mean absolute SHAP value for each feature is calculated by averaging the absolute SHAP value of all data points to provide an aggregated measure of feature importance across all instances, giving an overall importance magnitude of influence that a feature has, regardless of the direction of that influence. The AE duration was identified as the best feature contributing to the crack width, with mean absolute SHAP values of 2.95 and 3.08 in sensor 5 and sensor 6, respectively. The other AE features exhibited an inconsistent contribution to the expansion of crack width. 

**Figure 15:**
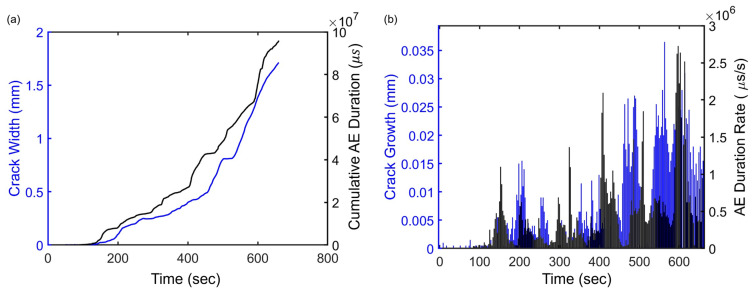
The relationship between AE duration and crack width using the sensor 5 data of slab 1. (**a**) AE cumulative duration and crack width and (**b**) AE duration rate per second and crack growth.

The linear relationships between all eight cumulative AE features and crack width data were quantified using the Pearson correlation coefficient, which is given by the following equation [54]:

(1) $$r_{feature}=\frac{\left(n\sum xy-\sum x\sum y\right)}{\sqrt{\left(\left(n{\sum x}^{2}-{\left(\sum x\right)}^{2}\right)\left(n{\sum y}^{2}-{\left(\sum y\right)}^{2}\right)\right)}}$$

where $r_{feature}$ is the Pearson correlation coefficient of a particular AE feature, n is the number of data points, x and y are two variables that represent an AE feature and crack feature, respectively. Among all eight AE features, the duration exhibits the highest cross-correlation coefficient of 0.9899. The cross-correlation coefficients for other features such as the AE frequency centroid and the AE initiation frequency are 0.8928 and 0.8673, respectively. The highest correlation coefficient value for AE duration represents the strongest degree of association between AE duration and crack width, as shown in Figure 15a, suggesting that AE duration has an exceptional predictive capability to crack growth compared to other factors. Figure 15b shows the AE duration rate and the crack width growth. The higher the crack width growth, the higher is the AE duration. The signal processing result can be explained by the fact that when an instantaneous crack grows a longer distance (i.e., larger crack width), it generates a source function with a longer duration, which causes the AE signal to have a longer duration. If the AE sensor is positioned in a mid-span, the cumulative AE duration can be tracked as an indicator of crack width. After the tensile cracks initiated at the mid-span, multiple additional cracks appeared, as illustrated in Figure 4c. The AE sensor was capable of detecting signals emitted by all these cracks. However, since the load was applied continuously, the widths of all the cracks increased simultaneously. Although the AE duration can still be used to correlate with the crack width measured at a specific position, it should be noted that the AE duration represents the cumulative effect of multiple cracks growing simultaneously within the proximity of the sensor.

## 5. Factors to Consider for Adapting the Models to Field Applications

The SHM applications to transfer from laboratory to field can be influenced by environmental factors and traffic noise. Since the AE method relies on high-frequency elastic waves, it is generally not affected by temperature changes. Concrete structures, being relatively more rigid than steel structures, tend to exhibit lower friction emissions. In a typical monitoring application, it is possible to determine the background noise characteristics during the initial stages of the measurement. When using the same type of AE sensor and similar sensor distance to the expected crack regions as reported in this study, the AE characteristics of cracks are expected to be similar. Therefore, the cluster features representing tensile and shear cracks can be transferred to the field. The secondary emissions representing noise cluster need to be identified at the initial stages of the field measurement. 

The AE method can be utilized for both global and local monitoring purposes. In the case of global monitoring, sensors are strategically placed across the structure to encompass the entire system. On the other hand, local monitoring is typically employed to track the progression of known defects or monitor critical regions. The relationship between AE duration and crack width proposed in this study specifically focuses on local monitoring.

## 6. Conclusions

In this study, the AE method was demonstrated as a viable SHM solution for detecting the initiation and progression of damage in BFPR-reinforced slabs. The main conclusions of this study are as follows:

- The load–deflection and load–strain curves were good indicators of damage initiation, as corroborated by visual inspection. Prior to the crack initiation, the load–deflection behaviors of the two slabs exhibited similar patterns, however, once the tensile cracks formed, the behaviors varied with respect to the reinforcement ratio. The AE behavior changed accordingly. The slab with the higher reinforcement ratio released fewer AE hits due to stiffer behavior. The reinforcement ratio plays an important role when the cumulative AE hits or energy curves are used to differentiate the damage progression. The relationship between AE features and the local strain was found to provide damage information in the absence of strain data. Mid-span sensor locations exhibited higher AE activity than support regions, as expected, due to their proximity to the tensile crack source.
- A methodology integrating cluster analysis and the k-nearest neighbor (K-NN) algorithm was presented for predicting the required principal component axes and clusters within data sets. The machine learning model’s validity was evaluated using multiple data sets to detect cracking types and progression toward failure. A distinct characteristic between tensile and shear cracks was shown to detect the failure progression. In a realistic environment where the load is not gradually increased to form damage, the machine learning model developed in this study can differentiate AE sources and identify the progression of cracking from tensile to shear. The accuracy of the K-NN model trained on the first slab was 99.2% in predicting three clusters (tensile crack, shear crack, and noise).
- SHAP (SHapley Additive exPlanations) analysis was shown as an effective signal processing tool to identify the most sensitive AE feature to the physical variable. The AE duration was determined as the feature related to the crack width. This is explained by the source function behavior that longer crack growth has a longer duration of source function that contributes to the duration of the AE signal as the AE signal is the convolution of source and medium transfer functions. The Pearson correlation coefficient of AE duration related to crack width achieves 0.9899. The approach is applicable for local monitoring to track the progression of known defects or to evaluate the damage state of critical regions.

In summary, the output of AE sensors at the mid-span of BFRP slabs could be used to monitor the initiation of tensile cracks, sudden changes in structural response due to major damage, and the progression of crack width (with AE duration). It is important to note that the absolute and cumulative AE values are influenced by the reinforcement ratio in addition to the AE sensor type, distance to AE source, and data acquisition variables. However, the machine learning model developed in this study reduces their influences by normalizing data and measuring the distances between cluster centers as a relative variable. Future investigation would involve analyzing the effectiveness and reliability of these AE time-frequency features in detecting and evaluating the damage in BFRP-reinforced concrete [55,56].

## Figures and Tables

**Table 1:** The slab specifications.

Slab #	Length × Width, m.	Bar Size	Reinforcement Spacing, cm.	Reinforcement Ratio
1	3.05 × 1.22	#5	15.24	0.773
2	3.05 × 1.22	#6	15.24	1.107

**Table 2:** Tensile properties of BFRP bars.

Bar Size	Young’s Modulus, MPa (ksi)	Ultimate Stress, MPa (ksi)
#5	59,805 (8674)	1392 (202)
#6	60,660 (8798)	1179 (171)

**Table 3:** The mean value of AE feature in each cluster obtained from sensor 5 of slab 1.

Cluster	AE Rise Time (μs)	AE Energy (aJ)	AE Duration (μs)	AE Amplitude (dB)	AE Average Frequency (kHz)	AE Peak Frequency (kHz)	AE Frequency Centroid (kHz)
1	188.4	4.9	572.9	44.6	32.0	38.6	107.5
2	127.44	3.9	137.4	45	43.1	98.9	120.2
3	1483.29	174.5	4652.9	61.5	28.7	46.2	84.1

**Table 4:** The summary of the training and testing sets as well as the classification results.

Training Set	Testing Set	Classification Results of Testing Set
Sensor 5, slab 1	Sensor 6, slab 1	Cluster 1—61.5% Cluster 2—26.3% Cluster 3—12.2%
	Sensor 5, slab 2	Cluster 1—60.1% Cluster 2—30.8% Cluster 3—9.1%

## Data Availability

The data presented in this study are available on request from the corresponding author.
